# miR-3656 expression enhances the chemosensitivity of pancreatic cancer to gemcitabine through modulation of the RHOF/EMT axis

**DOI:** 10.1038/cddis.2017.530

**Published:** 2017-10-19

**Authors:** Rui-Meng Yang, Ming Zhan, Sun-Wang Xu, Man-Mei Long, Lin-Hua Yang, Wei Chen, Shuai Huang, Qiang Liu, Jun Zhou, Jun Zhu, Jian Wang

**Affiliations:** 1 CNRS-LIA Hematology and Cancer, Sino-French Research Center for Life Sciences and Genomics, State Key Laboratory of Medical Genomics, Rui Jin Hospital, School of Medicine, Shanghai Jiao Tong University, Shanghai, China; 2 Department of Biliary-Pancreatic Surgery, Renji Hospital, School of Medicine, Shanghai Jiao Tong University, Shanghai, China; 3 Department of Pathology, Shanghai Ninth People’s Hospital, School of Medicine, Shanghai Jiao Tong University, Shanghai, China; 4 Department of Pathology, Renji Hospital, School of Medicine, Shanghai Jiao Tong University, Shanghai, China; 5 Université de Paris 7/INSERM/CNRS UMR 944/7212, Equipe Labellisée No. 11 Ligue Nationale Contre le Cancer, Hôpital St. Louis, Paris, France

## Abstract

The highly refractory nature of pancreatic cancer (PC) to chemotherapeutic drugs is one of the key reasons contributing to the poor prognosis of this disease. MicroRNAs (miRNAs) are key regulators of gene expression and have been implicated in a variety of processes from cancer development through to drug resistance. Herein, through miRNA profiling of gemcitabine-resistant (GR) and parental PANC-1 cell lines, we found a consistent reduction of miR-3656 in GR PANC-1 cells. miR-3656 overexpression enhanced the antitumor effect of gemcitabine, whereas silencing of miR-3656 resulted in the opposite effect. By performing mechanistic studies using both *in vitro* and *in vivo* models, we found that miR-3656 could target RHOF, a member of the Rho subfamily of small GTPases, and regulate the EMT process. Moreover, enforced EMT progression via TWIST1 overexpression compromised the chemotherapy-enhancing effects of miR-3656. Finally, we found significantly lower levels of miR-3656 and higher levels of RHOF in PC tissues compared with adjacent noncancerous pancreatic tissues, and this was also associated with poor PC patients’ prognosis. Taken together, our results suggest that the miR-3656/RHOF/EMT axis is an important factor involved in regulating GR in PC, and highlights the potential of novel miR-3656-based clinical modalities as a therapeutic approach in PC patients.

As one of the most common lethal malignancies, pancreatic cancer (PC) represents the fourth highest cause of cancer deaths worldwide, with a 5-year survival rate of only 7%.^1, 2^ Owing to our current inability to detect the disease in its early stages, most diagnosed patients miss the opportunity for curative surgery.^3^ Hence, chemotherapy has become critically important for the treatment of PC patients.^4^ Currently, gemcitabine-based chemotherapy forms the first-line treatment for PC,^5^ however, drug resistance, either intrinsic or acquired, compromises therapeutic efficacy and represents a significant challenge for the treatment of PC.^6, 7^ Although several characteristics such as, epithelial-to-mesenchymal transition (EMT) and the accumulation of cancer stem cells have been suggested as important contributors to PC chemoresistance,^8, 9^ the precise molecular mechanisms remain largely unknown.

MicroRNAs (miRNAs) are around 22 nucleotides in length and represent a group of evolutionarily conserved, single-stranded non-coding RNAs. Through binding to the 3′-untranslated regions (3′-UTRs) of target genes, they have been identified as key factors in modifying the biological behavior of various kinds of tumors.^10, 11^ Altered miRNAs have also been identified as an important mechanism leading to drug resistance in PC cells. For instance, elevated levels of the oncogenic miR-320c were found in PC cells following gemcitabine treatment,^12^ and reduced levels of miR-200 were also identified in gemcitabine-resistant (GR) PC cells.^9^ Moreover, the regulatory role of miRNAs in determining drug sensitivity appears to be fulfilled through multiple pathways, including cancer stem cells, multidrug resistance related-membrane transporters and the EMT process.^9, 13, 14^ However, the precise mechanism(s) of how miRNAs regulate the chemotherapeutic sensitivity of PC cells remain largely unknown and require further investigation.

EMT is a common feature of various types of tumors. During this process, cancer cells gradually lose expression of epithelial markers and instead, acquire the mesenchymal cell features required for further migration and invasion.^15^ Interestingly, recent evidence also suggests that the EMT process is tightly correlated with drug resistance.^16, 17^ Mouse PC models deficient in EMT-inducing transcription factors, such as TWIST1, Snail and ZEB1, reveal enhanced gemcitabine sensitivity and increased overall survival rates.^17, 18, 19^ Signaling pathways such as TGF-*β*, Wnt and Notch have also been reported to correlate with gemcitabine-induced EMT.^17, 18, 19, 20^ Nevertheless, exactly how the EMT process is regulated in PC is still not fully understood and elucidating the mechanisms involved could potentially provide clues for the development of novel PC therapies.

Herein, by comparing genome-wide miRNA expression profiling of GR and parental PC cell lines (combined with two previous databases), we identified common low-level expression of miR-3656 in GR PC cells. Indeed, alteration of miR-3656 expression levels could modulate the gemcitabine sensitivity of PC cells. Upon further molecular analysis, we demonstrated that reduced miR-3656 expression levels activated the EMT pathway through upregulation of RHOF, eventually causing drug resistance. Moreover, low miR-3656 and high RHOF expression was significantly associated with PC (compared with corresponding noncancerous pancreatic (CNP) tissues), and a tight association with poor prognosis was also identified. Taken together, our data suggest that miR-3656 is a novel factor in the regulation of PC gemcitabine sensitivity. Furthermore, our data provide new direction for the future development of potential molecularly targeted therapies in achieving improved therapeutic outcomes for PC patients.

## Results

### Identification of reduced miR-3656 levels in GR PC cell lines

**Figure 1:**
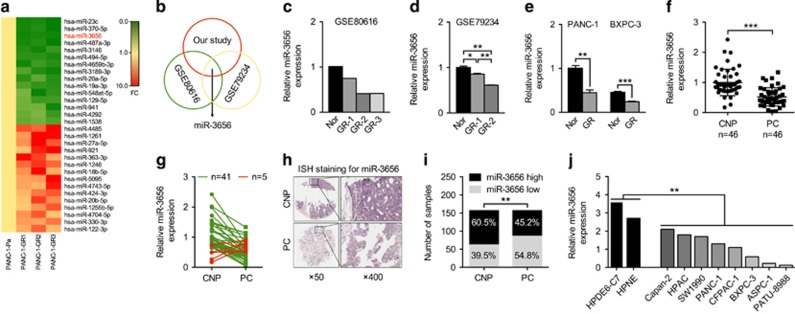
miR-3656 expression is reduced in GR pancreatic cell lines. (**a**) A heat map showing the top ranked differentially expressed miRNAs in the three clones of GR PANC-1 (PANC-1-GR) and parental PANC-1 (PANC-1-P) cells. The scale from 0 to 10 marks the intensity of differential regulation of miRNAs: low expression (green), mid expression (yellow) and high expression (red). (**b**) A Venn diagram showing the overlapped miRNAs associated with GR of PANC-1 cells from three different studies (our study, GSE80616 and GSE79234). (**c** and **d**) Comparison of miR-3656 expression between PANC-1-GR and PANC-1-P cells from GSE80616 and GSE79234 databases. (**e**) Validation of miR-3656 expression in the PANC-1 GR and BXPC-3 GR clones compared with their parental clone by qPCR. (**f**) Comparison of miR-3656 expression in 46 pairs of fresh PC and CNP tissues by qPCR. (**g**) Fold change of miR-3656 expression in 46 fresh PC and CNP tissues: downregulation (green) and upregulation (red). (**h**) Representative ISH staining analysis images of 157 PC and CNP FFPE tissues using an anti-miR-3656 probe. Images on the right are the enlarged versions. (**i**) The percentage of tissues displaying lower and higher miR-3656 levels in 157 PC and CNP FFPE samples by ISH staining. (**j**) Comparison of miR-3656 expression in eight PC cell lines with normal pancreatic epithelial cells by qPCR. *RNU6B* snRNA was used to normalize the qPCR results. Bar, S.E.M., *P<0.05; **P<0.01; ***P<0.001; Student’s *t-*test or *χ*^2^ test

To identify candidate regulators of chemoresistance in PC, we first established three independent clones of GR PANC-1 (PANC-1-GR) cells. MiRNA expression profiling of the three PANC-1-GR cells and the parental PANC-1 (PANC-1-P) cells was then performed. MiRNAs that were simultaneously upregulated or downregulated in the three PANC-1-GR clones compared with the PANC-1-P clone were selected for further analysis (Figure 1a, and Supplementary Table 1). When combined with two other expression profiles from gene expression omnibus (GEO) databases (GSE80616 and GSE79234) (also performed using PANC-1-GR and PANC-1-P cell lines), we found that miR-3656 was the only miRNA commonly reduced in all PANC-1-GR cell lines (Figures 1b and d). To confirm the lower miR-3656 expression levels, we used quantitative PCR (qPCR) assay to demonstrate low expression of miR-3656 in both PANC-1 and BXPC-3 GR cells compared with the parental cell lines (Figure 1e).

To investigate the possible involvement of miR-3656 in PC, we assessed its expression in a bank of 46 PC and CNP tissues. Our results showed clearly reduced levels of miR-3656 in PC compared with CNP tissues (Figure 1f), with 41/46 patients revealing uniformly reduced miR-3656 levels in PC tissues (Figure 1g). *In situ* hybridization (ISH) staining confirmed remarkably lower miR-3656 expression in 157 formalin-fixed paraffin-embedded (FFPE) PC tissue samples compared with their CNP tissues (Figures 1h and i). In addition, miR-3656 was also found to be reduced in various PC cell lines compared with normal pancreatic epithelial cell lines (HPDE6-C7 and HPNE) (Figure 1j).

### Reduced miR-3656 expression enhances PC cell GR through promoting the EMT process

**Figure 2:**
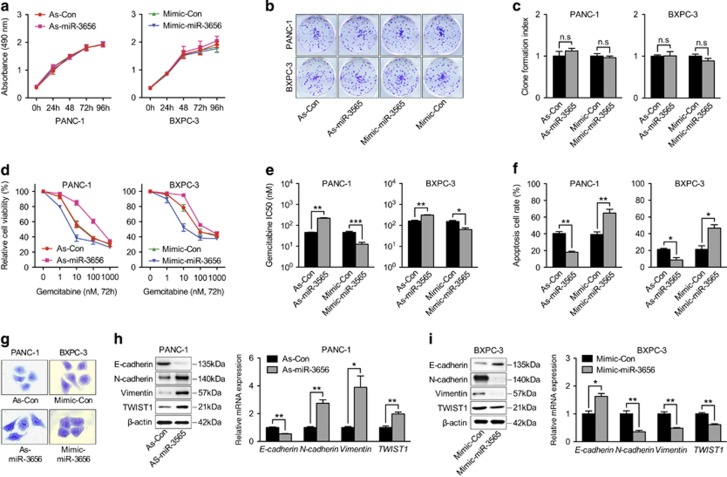
Altered miR-3656 expression in PC cell lines modifies sensitivity to gemcitabine. (**a**) MTS analysis of PANC-1 and BXPC-3 cells transfected with miR-3656 antisense (As-miR-3656), or negative control antisense (As-Con), miR-3656 mimic (Mimic-miR-3656), negative control mimic (Mimic-Con). (**b** and **c**) Representative images and statistical analysis of colony formation index among PANC-1 and BXPC-3 cells transfected with miR-3656 mimic, antagomir, or negative control. (**d** and **e**) MTS assay and the corresponding IC50 of gemcitabine measured in PANC-1 and BXPC-3 cells transfected with miR-3656 mimic, antagomir, or negative control. (**f**) Quantification of Annexin V/PI-positive apoptotic cells in PANC-1 and BXPC-3 cells transfected with miR-3656 mimic, antagomir or negative control after gemcitabine treatment (10 nM) via flow cytometric analysis. (**g**) Representative images of crystal violet staining showing the morphological changes of PANC-1 cells transfected with miR-3656 antisense or control antisense and BXPC-3 cells with miR-3656 mimic or control mimic. (**h** and **i**) EMT-related marker expression in PANC-1 cells transfected with miR-3656 antisense or control antisense, and in BXPC-3 cells with miR-3656 mimic or control mimic addition shown by western blotting and qPCR. *GAPDH* was used to normalize the qPCR results, and *β*-actin was used as a loading control in western blots. All n=3; bar, S.E.M., n.s, not significant, *P<0.05; **P<0.01; ***P<0.001; Student’s *t*-test

To further explore the biological role of miR-3656, antisense-miR-3656 and mimic-miR-3656 were used in PANC-1 and BXPC-3 cells, respectively, for modulating miR-3656 expression. We found that neither increasing miR-3656 expression using the miR-3656 mimic, nor reducing miR-3656 expression via antisense-miR-3656 transfection influenced the proliferation rate of PANC-1 and BXPC-3 cells (Figure 2a). Similarly, colony-forming ability was assayed following modulation of miR-3656 levels in both PANC-1 and BXPC-3 cell lines, and also showed no obvious differences (Figures 2b and c). We then examined the effect of modulating miR-3656 levels on the gemcitabine potency toward both PANC-1 and BXPC-3 cell lines. Various concentrations of gemcitabine were used and cell viabilities were analyzed 72 h after treatment. Interestingly, treatment with the miR-3656 mimic enhanced the cytotoxicity of gemcitabine in PANC-1 and BXPC-3 cells with remarkably reduced IC50’s observed (Figures 2d and e). In contrast, reducing miR-3656 expression levels using antisense-miR-3656 treatment conferred a higher degree of GR in both PANC-1 and BXPC-3 cell lines, with obviously increased IC50 values (Figures 2d and e). Flow cytometry analysis of Annexin V/PI-positive apoptotic cells with modified miR-3656 expression in PANC-1 and BXPC-3 cells further confirmed the influential role of miR-3656 on the potency of gemcitabine treatment (Figure 2f).

Intriguingly, PANC-1 cells with reduced miR-3656 levels showed an increase in the number of cells with elongated mesenchymal-like morphology and fewer cell–cell junctions, whereas cells with epithelial-like morphology were elevated in BXPC-3 cell cultures treated with the miR-3656 mimic (Figure 2g). The phenotypic conversion of epithelial cells to mesenchymal cells, named EMT, has been identified as a key process in the malignant transformation of multiple cancers. Concomitantly, although the epithelial-related marker E-cadherin was reduced in PANC-1 cells transfected with antisense-miR-3656, proteins involved in the mesenchymal transition such as, N-cadherin, Vimentin and TWIST1 were increased (Figure 2h). qPCR assay confirmed the reduced mRNA expression of E-cadherin and elevation of N-cadherin, Vimentin and TWIST1 upon lowering of miR-3656 levels (Figure 2h). Consistently, enforced expression of miR-3656 in BXPC-3 cells manifested the opposite effect, with increased epithelial markers and reduced mesenchymal markers (Figure 2i). Alongside the role of EMT in promoting tumor invasion, increasing evidence suggests that this process may also be involved in modulating the chemosensitivity of cancer cells. To validate the hypothesis that miR-3656’s chemomodulatory role was coupled to the EMT process, TWIST1 (an important EMT-promoting transcription factor) was next overexpressed together with miR-3656. Interestingly, TWIST1 overexpression abolished the chemotherapeutic-enhancing effect of miR-3656 as shown by increased cell viability and less potent IC50 upon gemcitabine treatment (Supplementary Figures 1a and 1b). In addition, a lower percentage of apoptotic cells were observed upon gemcitabine treatment when TWIST1 was overexpressed with miR-3656 in PANC-1 and BXPC-3 cells (Supplementary Figure 1c). In conclusion, our results suggest that increased miR-3656 sensitizes PC cells to gemcitabine, and that this effect likely relies on reversing the EMT process.

### miR-3656 targets the 3′-UTR of *RHOF* and suppresses its expression

**Figure 3:**
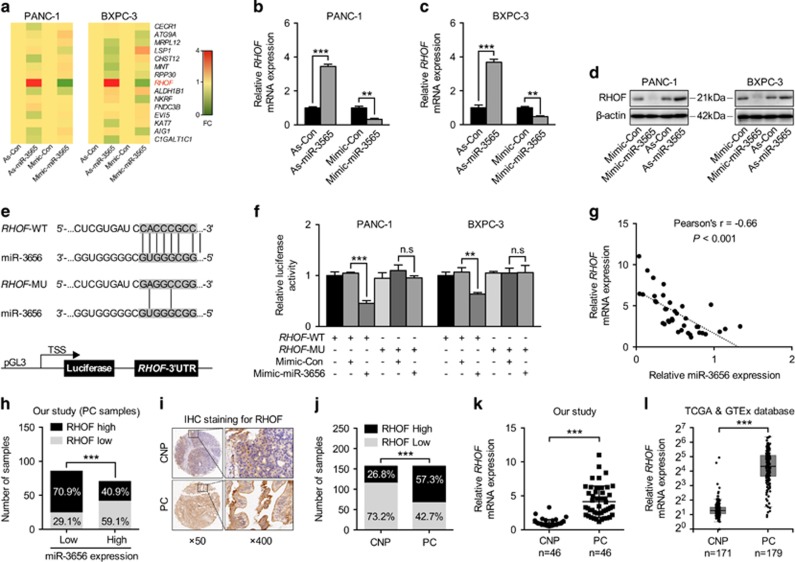
miR-3656 directly targets *RHOF* and represses its expression. (**a**) A heat map of the expression changes of 15 candidate genes predicted to be targets of miR-3656 in PANC-1 and BXPC-3 cells transfected with miR-3656 mimic, antagomir or negative control. The scale from 0 to 4 marks the intensity of differential regulation of mRNAs: low expression (green), mid expression (yellow), and high expression (red). (**b**–**d**) The expression of RHOF in PANC-1 or BXPC-3 cells transfected with miR-3656 mimic, antagomir or negative control as examined by qPCR and western blotting. (**e**) Schematic diagram showing the predicted binding sequence between *RHOF* and miR-3656. The wild-type (WT) and mutant (MU) 3′-UTR of *RHOF* were cloned into luciferase reporter constructs. (**f**) The relative luciferase activity of either WT or MU 3′-UTR of *RHOF* reporter with miR-3656 mimic addition in PANC-1 and BXPC-3 cells. (**g**) The relationship of the expression between miR-3656 and *RHOF* in 46 fresh PC tissues via qPCR assay. (**h**) Calculated percentages of RHOF expression in either miR-3656 low or high expression groups from 157 PC FFPE samples. (**i** and **j**) Representative images and semiquantitative analyses of IHC staining for RHOF in 157 PC and CNP FFPE tissues. (**k**) qPCR examination of *RHOF* levels in 46 PC and CNP tissues. (**i**) TCGA and GTEx database data showing the relative *RHOF* expression levels in PC and CNP tissues. *RNU6B* snRNA was used to normalize the qPCR results. All *n*=3; bar, S.E.M., n.s, not significant, *P<0.05; **P<0.01; ***P<0.001; Student’s *t*-test or *χ*^2^ test

Through binding to the 3′-UTR of target genes, miRNAs specifically regulate the expression of various genes. Combining three prediction models, we identified 15 potential candidate target genes for miR-3656, and analyzed their expression levels following either increased or decreased expression of miR-3656 in both PANC-1 and BXPC-3 cell lines (Figure 3a). Among these, we found that *RHOF* was the only gene showing the same modulation in both PANC-1 and BXPC-3 cells, namely, increased *RHOF* with antisense-miR-3656 transfection and reduced *RHOF* with miR-3656 mimic treatment (Figures 3a and c). Consequently, we then assayed RHOF protein levels upon modulation of miR-3656 expression. As expected, an inverse relationship was identified between RHOF and miR-3656 in both PANC-1 and BXPC-3 cell lines (Figure 3d). To further investigate any direct regulatory role for miR-3656 on *RHOF* expression, the predicted complementary pairing region of the 3′-UTR of miR-3656 with *RHOF*-WT (5′-CACCCGCC-3′) was mutated into *RHOF*-MU (5′-GAGGCCGG-3′) and subsequently cloned into a luciferase reporter vector (Figure 3e). Although the addition of the miR-3656 mimic repressed *RHOF*-WT luciferase reporter activity in both PANC-1 and BXPC-3 cell lines, this effect disappeared when using the *RHOF*-MU reporter (Figure 3f). Importantly, inverse relationships between miR-3656 and *RHOF,* as well as its protein levels, were also detected in our 46 fresh PC samples (Pearson’s r=–0.66, *P*<0.001) and 157 FFPE PC samples (OR=0.28, *P*<0.001) (Figures 3g and h).

To our knowledge, the involvement of RHOF in PC has never been previously investigated. Our data are the first to analyze the expression of RHOF in PC and CNP human tissue samples. A clear increase in RHOF protein expression was observed in 157 FFPE PC samples compared with the CNP tissues using an immunohistochemistry (IHC) staining assay (Figures 3i and j). qPCR analysis also revealed elevated *RHOF* expression in the 46 fresh PC samples compared with their CNP tissues (Figure 3k). Consistent with our results, both the cancer genome atlas (TCGA) and genotype-tissue expression project (GTEx) databases also show significantly increased *RHOF* in PC tissue samples (Figure 3I).

### miR-3656 reduces RHOF expression and results in increased PC cell gemcitabine sensitivity

**Figure 4:**
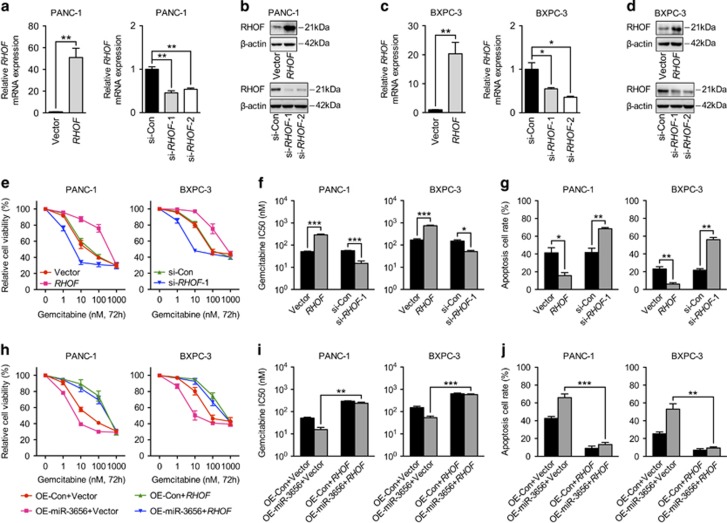
miR-3656 sensitizes PC cells to gemcitabine is RHOF-dependent. (**a-d**) qPCR and western blotting assays confirming the overexpression or knock down of *RHOF* in both PANC-1 and BXPC-3 cells. (**e** and **f**) MTS assay and the gemcitabine IC50 measurements in PANC-1 and BXPC-3 cells transfected with the *RHOF* construct, *RHOF* siRNA or negative control. (**g**) The percentage of apoptotic cells in PC cells transfected with the *RHOF* construct, *RHOF* siRNA or negative control using flow cytometric analysis. (**h** and **i**) Comparison of cell viability and the IC50 of gemcitabine in PANC-1 and BXPC-3 cells with miR-3656 overexpression alone or miR-3656 overexpression combined with *RHOF* construct transfection. (**j**) Apoptosis assays in PANC-1 and BXPC-3 cells overexpressing miR-3656 alone or miR-3656 overexpression combined with *RHOF* construct transfection. *GAPDH* was used to normalize the qPCR results, and *β*-actin was used as a loading control in western blotting assays. All *n*=3; bar, S.E.M., *P<0.05; **P<0.01; ***P<0.001; Student’s *t-*test

In order to confirm the possible involvement of RHOF in regulating chemotherapeutic efficacy, we first modified the expression levels of RHOF in PANC-1 and BXPC-3 cell lines using an *RHOF* overexpression vector or siRNA, respectively. qPCR and western blotting analysis confirmed RHOF overexpression and knock down (Figures 4a and d). Indeed, elevated RHOF expression in both PANC-1 and BXPC-3 cell lines increased their viability and decreased the potency of gemcitabine, whereas reduced RHOF manifested the opposite effects (Figures 4e and f). Apoptotic cells analyzed by flow cytometry analysis also confirmed the weakened cytotoxic effect of gemcitabine against *RHOF*-overexpressing cells and enhanced cell killing effect in *RHOF* low-expressing cells (Figure 4g). To establish whether RHOF is involved in mediating the chemo-modifying effect of miR-3656, we then overexpressed *RHOF* and miR-3656 simultaneously in both PANC-1 and BXPC-3 cell lines. Our data above suggested that single overexpression of miR-3656 could enhance the gemcitabine sensitivity, as shown by reduced IC50’s and increased numbers of apoptotic cells. This effect, however, when analyzed in cells co-expressing miR-3656 and *RHOF* was largely weakened (Figures 4h and j). Our results therefore suggest that RHOF may be a strong candidate target gene of miR-3656 with a significant influence on the regulation of chemotherapeutic efficacy.

### miR-3656/RHOF targets the EMT pathway to modulate chemotherapeutic efficacy

**Figure 5:**
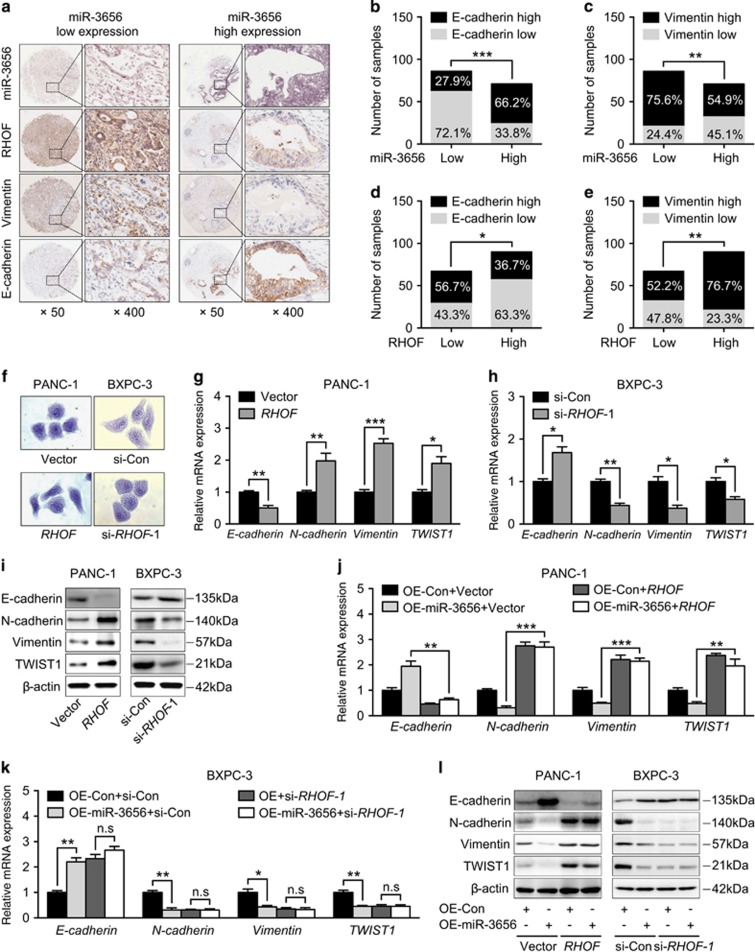
miR-3656 targeting of RHOF modulates the EMT process in PC. (**a**) Representative images showing the associations between miR-3656 expression, RHOF and EMT markers (Vimentin and E-cadherin) in 157 PC FFPE samples. (**b–e**) The percentage scoring of EMT marker (Vimentin and E-cadherin) expression in either miR-3656 low or high expression groups, and in either RHOF low or high expression groups from 157 PC FFPE samples. (**f**) Morphological changes resulting from RHOF overexpression in PANC-1 cells and *RHOF*-deficient BXPC-3 cells via crystal violet staining. (**g-i**) Examination of EMT marker (E-cadherin, N-cadherin, Vimentin and TWIST1) expression in *RHOF*-overexpressing PANC-1 cells and RHOF low expressing BXPC-3 cells by qPCR and western blotting assay. (**j**) Comparison of EMT marker (E-cadherin, N-cadherin, Vimentin and TWIST1) expression between miR-3656 single overexpressing and miR-3656/*RHOF* co-overexpressing PANC-1 cells. (**k**) Expression changes of EMT markers among si-*RHOF*, miR-3656 overexpressing and si-*RHOF*/miR-3656 overexpressing BXPC-3 cells via qPCR. (**l**) Western blotting assay of (**j** and **k**). *GAPDH* was used to normalize the qPCR results, and *β*-actin was used as a loading control in western blots. All *n*=3; bar, S.E.M., *P<0.05; **P<0.01; ***P<0.001; Student’s *t*-test or *χ*^2^ test

Increasing evidence suggests that EMT transformed cells contribute significantly to chemoresistance through mechanisms such as, reduced cell proliferation, apoptotic resistance and increased numbers of cancer stem cells. We found that samples from 157 PC tissues with lower levels of miR-3656 were often associated with higher levels of RHOF and Vimentin (mesenchymal marker) and lower levels of E-cadherin (epithelial marker) expression (Figure 5a). Statistical analysis further confirmed this inverse relationship between miR-3656 and mesenchymal markers, and the positive correlation between RHOF and mesenchymal markers (Figures 5b and e).

RHOF, a member of the Rho GTPase family, is an important regulator of cell adhesion and migration. Cells undergoing EMT require a series of morphological and molecular changes and Rho GTPase members can promote EMT progression through directly increasing the invasive potential of cancer cells. The involvement of RHOF in cancer development has been reported, but whether it is also implicated in regulating EMT has never been investigated. To address this question, we then examined EMT-related phenotypes in both PANC-1 and BXPC-3 cell lines with modified RHOF expression. Enforced RHOF expression manifested increased PANC-1 cells with mesenchymal morphology (Figure 5f). Moreover, qPCR assay confirmed reduced epithelial marker (E-cadherin) and increased mesenchymal markers (N-cadherin, Vimentin, TWIST1) accompanied with *RHOF* overexpression (Figure 5g). In contrast, an epithelial-like morphology was induced in *RHOF*-deficient BXPC-3 cells (Figure 5f) and correspondingly, increased epithelial marker (E-cadherin) and reduced mesenchymal markers (N-cadherin, Vimentin, TWIST1) were also detected (Figure 5h). Further western blotting analysis of EMT-related markers confirmed the ability of RHOF to induce a mesenchymal phenotype and inhibit the epithelial phenotype in both PANC-1 and BXPC-3 cells (Figure 5i). Finally, we examined whether miR-3656 modulated the EMT process through regulation of RHOF. Indeed, *RHOF* overexpression faithfully restored the EMT-reversing effect of miR-3656 (Figures 5j and I). Furthermore, the ability of miR-3656 to regulate the EMT process was abolished in *RHOF*-deficient cells (Figures 5k and l). In conclusion, our results show that miR-3656 has a modulatory role on the RHOF/EMT axis, which in turn directly influences the sensitivity of PC cells to chemotherapy.

### miR-3656 enhances the chemotherapeutic effect of gemcitabine in nude mouse xenograft models

**Figure 6:**
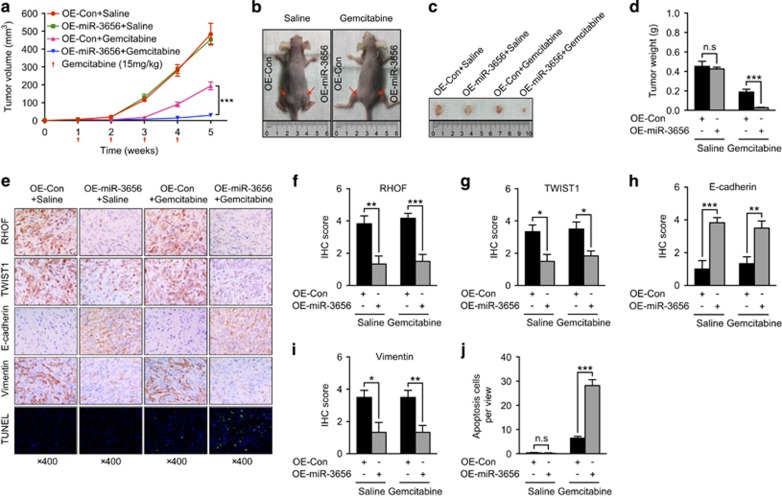
miR-3656 expression modulates the gemcitabine sensitivity of PC cells *in vivo*. (**a**) Tumor growth curves of PANC-1 cells transfected with a miR-3656 construct or empty vector and treated with gemcitabine or saline. (**b** and **c**) Representative images showing the tumors formed in the four groups, including control overexpression + saline (OE-Con + Saline), miR-3656 overexpression + saline (OE-miR-3656 + Saline), control overexpression + gemcitabine (OE-Con + Gemcitabine), miR-3656 overexpression + gemcitabine (OE-miR-3656 + Gemcitabine), at the 5th week after subcutaneous transplantation or when mice were killed. (**d**) Comparison of the mean tumor weights of the four groups. (**e**) Representative IHC images for RHOF, TWIST1, E-cadherin and Vimentin or TUNEL staining among the four tumor xenografts groups. (**f**-**j**) Statistical comparisons of RHOF, TWIST1, E-cadherin, Vimentin expression and apoptotic cell numbers among the four tumor xenografts groups. All *n*=6; bar, S.E.M., n.s, not significant, *P<0.05; **P<0.01; ***P<0.001; Student’s *t*-test

In order to explore the influence of miR-3656 on the efficacy of chemotherapy *in vivo,* we performed studies using mouse xenograft models. In mice, PANC-1 tumors transfected with miR-3656 grew at the same rate as those transfected with control plasmids (Figures 6a and d). However, when animal groups were treated with gemcitabine, tumors overexpressing miR-3656 grew at a significantly slower rate, with lower tumor volumes and weight, compared with the control group treated with gemcitabine (Figures 6a and d). IHC staining of miR-3656 tumor sections confirmed reduced RHOF, increased epithelial marker E-cadherin and reduced expression of the mesenchymal markers TWIST1 and Vimentin (Figures 6e and i). Moreover, transferase dUTP nick end-labeling (TUNEL) analysis of apoptotic cell numbers further confirmed the improved antitumor effect of gemcitabine in tumors with miR-3656 overexpression (Figures 6e and j). Collectively, our xenograft studies provide further evidence that miR-3656 overexpression enhances the chemotherapeutic effect of gemcitabine, which likely relies on its role in counteracting the RHOF-mediated EMT process.

### Reduced miR-3656 and increased RHOF expression correlate with poor PC patient prognosis

**Figure 7:**
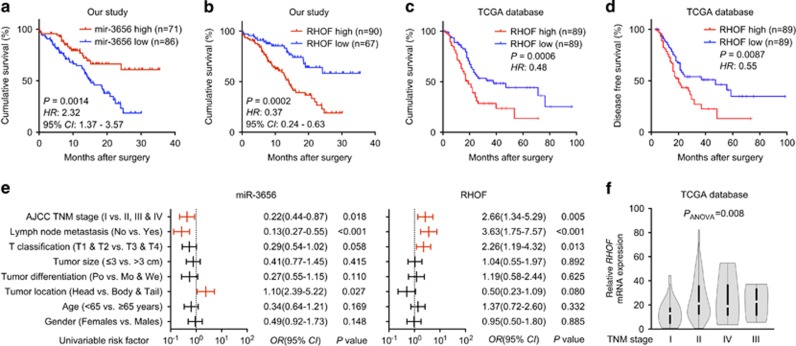
Low miR-3656 and high RHOF expression in PC tissues correlates with poor patient prognosis. (**a** and **b**) Kaplan–Meier analysis of the correlations between miR-3656 or RHOF expression and prognosis in 157 PC patients. (**c** and **d**) The correlation between RHOF expression and cumulative survival rate or disease-free survival rate of 178 PC patients from the TCGA database analyzed by Kaplan–Meier analysis. Statistical significance was determined using the log-rank test. (**e**) Comparison of the TNM stages, lymph node metastasis, T classification, tumor size, tumor differentiation, tumor location, age and gender among 157 PC patients according to the expression levels of either miR-3656 or RHOF. Statistical significance was performed using *χ*^2^ test. (**f**) A correlation was identified between *RHOF* and the TNM stage of PC patients within the TCGA database data using ANOVA analysis

Given the lower miR-3656 and higher RHOF expression levels detected in our PC compared with CNP samples, we also explored the correlations between patients’ cumulative survival rate and miR-3656 or RHOF expression. Although patients with higher miR-3656 expression had better outcomes than those with lower miR-3656 levels (Figure 7a), reduced RHOF expression appeared to further improve the prognosis of these PC patients (Figure 7b). Consistently, analysis of data from the TCGA database also confirmed both reduced cumulative survival rate and disease-free survival rates among those patient samples with higher RHOF levels (Figures 7c and d). Finally, we also performed an analysis of the correlations between patients’ clinical characteristics and miR-3656 or RHOF expression in our 157 PC patients. We identified associations between miR-3656 and TNM stage, lymph node metastasis and tumor location, whereas for RHOF, expression was associated with TNM stage, lymph node metastasis and T classification. The association between RHOF expression and TNM stage was also confirmed within data from the TCGA database (Figure 7f).

## Discussion

The highly malignant nature of PC, together with its difficult detection and inherent chemoresistance all contribute to the poor prognosis of PC patients.^21^ Accordingly, a better understanding of the molecular mechanisms underlying these aspects of the disease is urgently needed to enable design of targeted therapies to improve the treatment of PC. In this study, through comparing differential miRNA expression profiles between GR PC cells and parental PC cells, we built on previous database findings and identified miR-3656 as being expressed at significantly lower levels in GR PC cells. Through further mechanistic investigations using both *in vitro* and *in vivo* models, we found that miR-3656 targeted the *RHOF* gene to regulate EMT progression, which in turn contributed to PC chemoresistance. Tight correlations were identified among miR-3656, RHOF and EMT markers in both PC patients’ samples and mouse tumor xenograft models. Finally, but importantly, we also found that the expression of miR-3656 and RHOF correlated well with PC patients’ prognosis. Collectively, our study provides new clues for the design of future drugs to enhance the sensitivity of PC through targeting of the miR-3656/RHOF/EMT axis.

miRNAs are important regulators during the development of various kinds of tumors.^10, 11^ Aberrant miRNA expression in the serum and cancer tissues of PC patients has been found to be tightly correlated with tumor stage, drug resistance and patients’ survival.^9, 22^ In our study, we found reduced expression of miR-3656 in both GR PC cell lines and PC tissues. Moreover, decreased levels of miR-3656 also correlated with poor PC patient prognosis. Reduced miR-3656 levels have also been found in breast cancer patients’ tissues and peripheral blood,^23^ however, exactly how miR-3656 regulates the biological behavior of PC is still not known. Altering the miR-3656 levels within PC cells *in vitro* revealed no obvious effects on proliferation, but specifically influenced their sensitivity to gemcitabine. Importantly, we observed an interesting phenomenon in that PC cells with reduced miR-3656 expression levels acquired a mesenchymal-like phenotype including, elongated fibroblastoid shape, high expression of Vimentin and N-cadherin, and low expression of E-cadherin. In contrast, PC cells with increased miR-3656 expression showed induction of epithelial marker expression. More importantly, enforced EMT through TWIST1 overexpression in PC cells neutralized the gemcitabine sensitizing function of miR-3656. Increasing evidence has confirmed the critical importance of EMT not only in cancer progression, but also for resistance to chemotherapeutic drugs.^16, 17^ It has been shown that mesenchymal-type cancer cells with increased expression of genes related to the processes of invasion and metastasis often show resistance to drug treatment.^24^ Epithelial cells on the other hand, show less invasive and metastatic potential and are often more sensitive to chemotherapies.^16, 17^ The regulatory role of miRNAs on the EMT process could potentially be performed via several routes, such as direct targeting of EMT transcription factors or components of cell architecture required for EMT progression.

Through our miRNA profiling work, we identified the miR-3656 downstream candidate target gene, *RHOF,* and showed direct regulation by miR-3656. Importantly, we showed significant elevations in *RHOF* expression in PC tissues of our own samples and also of those within the TCGA and GTEx databases. Elevated RHOF expression in PC cell lines enhanced their resistance to gemcitabine cytotoxicity and led to EMTs. The RHOF protein belongs to the Rho subfamily of small GTPases, which are important regulators of many fundamental cellular processes such as epithelial adhesion, cell polarity, cell migration and membrane trafficking.^25^ The involvement of Rho GTPases, such as Rac1, Rnd1 and RhoC in cancer progression have been detailed elsewhere.^26, 27, 28, 29^ A reliance on elevated Rho GTPase expression has often been suggested for cells undergoing EMT.^26, 27, 28^ The involvement of RHOF in several types of cancers has also been reported. For example, neoplastic cells and transformed lymphomas exhibit elevated RHOF expression compared with their benign counterparts.^30^ It has also been mentioned that proteins of the RHOF subgroup have unique abilities relating to the regulation of dynamic cytoskeletal reorganization. Our study provides new data indicating that RHOF-modulated EMT is involved in counteracting drug treatment in PC cells.

EMT has long been well known for its role in inducing tumor metastasis. Indeed, alongside an improved antitumor effect observed with miR-3656 overexpression, miR-3656 and RHOF were also found to correlate with TNM stages and lymph node metastasis in PC patients. Lately, it has been recognized that malignant tumor properties such as metastasis, immune evasion and chemoresistance are tightly correlated and can in fact influence each other.^31^ Ongoing studies in our lab are focused on exploring whether miR-3656 may also be involved in regulating PC cells’ invasive and metastatic abilities.

In conclusion, our studies identify the novel miRNA, miR-3656, as a key modulator of PC chemosensitivity. This effect likely relies on its role in repression of the RHOF-mediated EMT process. These results provide new areas of research for developing modalities to enhance chemotherapeutic effects in PC.

## Materials and methods

### Clinical specimens

This study was approved by the Ethical Committee of Renji hospital, School of Medicine, Shanghai Jiao Tong University. All of the subjects were provided with written informed consent before enrollment. A total of 157 pairs of FFPE PC and CNP tissue samples used in this study were obtained from the Department of Pathology at Renji hospital. All PC patients underwent surgical resection without any neoadjuvant therapies, and samples were collected at the department of Biliary-Pancreatic Surgery of Renji hospital from January 2013 to September 2016. In addition, fresh PC tissues and CNP tissues were archived from 46 of 157 PC patients. All PC patients were retrospectively followed up until December 2016. The definition of postoperative survival is the interval between the dates of surgery and last follow up or death.

### Cell culture

Human pancreatic ductal epithelial cell lines HPDE6-C7 and HPNE were obtained from the American Type Culture Collection (ATCC, Manassas, VA, USA). Human PC cell lines Capan-2, HPAC, SW1990, PANC-1, CFPAC-1, BXPC-3, ASPC-1, PATU-8988 were all preserved in the lab of biliary-pancreatic surgery at Renji hospital. All cell lines were cultured in RPMI-1640 (Gibco, Grant Island, NY, USA), which was supplemented with 10% fetal bovine serum in a humidified atmosphere of 5% CO_2_ at 37 °C. GR PC cells were selected by continuous treatment of PANC-1 and BXPC-3 cells with 1000 nM gemcitabine (Selleck, Houston, TX, USA), when the confluence of cells reached 50% resulting in subclones resistant to gemcitabine. Three independent GR clones of PANC-1 and BXPC-3 cells were established, respectively.

### Cell transfection

The miR-3656 mimic (Mimic-miR-3656) and nonspecific mimic control (Mimic-Con), miR-3656 antisense (As-miR-3656) and nonspecific antisense control (As-Con), *RHOF* siRNA (si-*RHOF*) and negative control siRNA (si-Con) were all purchased from GenePharma (Shanghai, China), and were transfected into PC cell lines using Lipofectamine 2000 reagent (Invitrogen, Carlsbad, CA, USA) according to the manufacturer’s instructions. The human miR-3656 construct was generated by insertion of the coding sequence (CDS) of miR-3656 into pCDH-CMV-MCS-EF1-copGFP (System Biosciences, Palo Alto, CA, USA). Lentivirus packaging was performed in HEK293FT cells and then infected PANC-1 cells with 1 × 10^6^ recombinant lentivirus-transducing units in the presence of 4 *μ*g/ml polybrene (Sigma, St. Louis, MO, USA). Stable miR-3656 overexpression PANC-1 cells were obtained by 2 *μ*g/ml puromycin (Gibco) selection. *RHOF* and *TWIST1* overexpression vectors were generated by insertion of their CDS into a pcDNA 3.1 vector (Invitrogen). PANC-1 and BXPC-3 cells were transfected with the recombinant vector or empty vector using Lipofectamine 2000 reagent (Invitrogen) at 60-70% confluence.

### Cloning efficiency, cell proliferation, cell viability and cell apoptosis assays

For evaluation of colony formation capacity, PC cells transfected with different oligonucleotides were plated in six-well plates at a density of 500 cells per well and then incubated in the plate for 2 weeks until colonies were visible. The cell colonies were fixed for 10 min with 100% methanol and stained with 0.1% crystal violet. For cell proliferation analysis, 5000 PC cells were plated on 96-well plates. After transfection, absorbance at 490 nm was measured every 24 h for 4 days using MTS reagent (Promega, Madison, WI, USA) in a Synergy 2 (Biotek, Winooski, VT, USA) plate reader. For cell viability analysis, the transfected PANC-1 and BXPC-3 cells were treated with gemcitabine at concentrations of 0, 1, 10, 100 or 1000 nM and cultured in a 96-well plate for 72 h. The cell viability was measured by MTS assay as described previously.^14^ For cell apoptosis assays, the transfected PANC-1 and BXPC-3 cells were treated with gemcitabine (10 nM) and cultured in six-well plates for 72h. The PC cells were then stained with FITC-conjugated Annexin V (BD Biosciences, Heidelberg, Germany) and propidium iodide (5 mg/ml), and analyzed by fluorescence-activated cell sorting analysis according to our published protocols.^14^

### miRNA expression profiling

Total miRNAs were isolated from the parental PANC-1 clone and three independent GR PANC-1 clones using miRNeasy Mini Kit according to the instructions of the manufacturer (Qiagen, Valencia, CA, USA). The miRNA expression profiling was performed by using the miRCURY LNA expression array (Exiqon, Vedbaek, Denmark).

### qPCR analysis

Total RNAs were extracted from tissues or cells using TRI reagent (Sigma) or miRNeasy Mini Kit (Qiagen), and the cDNA’s were transcribed through Reverse Transcriptase M-MLV kit (Invitrogen) or Taqman microRNA Reverse Transcription kits (Thermo Fisher Scientific, Dreieich, Germany) according to the manufacturer’s instructions. qPCR was performed using the SYBR Premix Ex Taq (Takara, Shiga, Japan) or Taqman Gene Expression master mix (Thermo Fisher Scientific) in Applied Biosystems ViiATM 7 Real-Time PCR System (Applied Biosystems, Foster City, CA, USA). Data were calculated with 2^−ΔΔCT^ method and normalized to *GAPDH* mRNA land *RNU6B* snRNA levels. Primers for miR-3656 and *RNU6B* were from Thermo Fisher Scientific. The other primers were purchased from Sangon Biotech (Shanghai, China) and the sequences are listed in Supplementary Table 2.

### Western blotting

Total proteins were extracted from PC cells using RIPA Lysis and Extraction Buffer (Thermo Fisher Scientific). Protein concentrations were measured using a BCA Protein Assay Kit (Thermo Fisher Scientific). Standard western blotting techniques, and the Bio-Rad ChemiDoc MP imaging system (Hercules, CA, USA) were used according to the procedure described previously.^14^ The primary antibodies used were as follows: RHOF (1 : 1000, ab101349, Abcam, Cambridge, UK), TWIST1 (1 : 500, ab50887, Abcam), E-cadherin (1 : 1000, 3195, CST, Danvers, MA, USA), N-cadherin (1 : 1000, 14215, CST), Vimentin (1 : 1000, 5741, CST) and *β*-actin (1:2000, A5316, Sigma).

### ISH, IHC and TUNEL assay

The ISH, IHC and TUNEL staining of FFPE tissues were performed as described previously.^14^ The primary antibodies used in IHC were as follows: RHOF (1 : 200, ab101349, Abcam), TWIST1 (1 : 200, PA5-49688, Invitrogen), E-cadherin (1 : 400, 3195, CST), Vimentin (1 : 100, 5741, CST). Semiquantitative scoring of ISH and IHC was based upon the staining intensity (I: negative, 0; weak, 1; moderate, 2; intense, 3) and the percentage of positive-staining cells (P: 0–5%, scored 0; 6–35%, scored 1; 36–70%, scored 2; and >70%, scored 3) to obtain a final score (Q) defined as the product of I × P. Low expression was defined as Q<4 and high expression with Q ≥ 4. Two senior pathologists performed the scorings independently in a blinded manner.

### Dual-luciferase reporter assay

The 3′-UTR of *RHOF* containing the predicted miR-3656-binding site was amplified by PCR and then cloned into a pmirGLO Dual-Luciferase miRNA Target Expression Vector (Promega) to construct the wild-type reporter vector of *RHOF*-3′-UTR. The mutant reporter vector of *RHOF*-3′-UTR was generated using a site-directed mutagenesis kit from Fast Mutagenesis System (TransGen Biotech, Beijing, China) based on the wild-type reporter vector of *RHOF*-3′-UTR. The protocol used for transfection and measurement of luciferase activity has been described previously.^14^

### Bioinformatics

The target genes of miR-3656 were predicted by three computer-aided algorithms,^32, 33^ namely TargetScan Release 7.0 (http://www.targetscan.org/vert_71/), Microcosm Targets (http://www.ebi.ac.uk/enright-srv/microcosm/htdocs/targets/v5/) and miRNAMap 2.0 (http://mirnamap.mbc.nctu.edu.tw). The target genes were selected only when they were positive in all three algorithms using miRTarBase (http://mirtarbase.mbc.nctu.edu.tw/).^34^ The data sets of GSE80616 and GSE79234 were downloaded from the public source GEO data repository.^35^ The *RHOF* expression data for PC and the corresponding prognostic data were downloaded from TCGA, which were processed and analyzed by GEPIA, a web server for cancer and normal gene expression profiling and interactive analyses.^36^

### Animal studies

The Animal Research Committee of Renji hospital and Shanghai Jiao Tong University approved all experimental protocols and surgical procedures. Twelve BALB/c nude mice (SLARC Inc., Shanghai, China; 4-week-old; 15–20 g) were subcutaneously inoculated in the right and left hind footpads with 2 × 10^6^ PANC-1-OE-Con and PANC-1-OE-miR-3656 cells, respectively. Seven days after inoculation, mice were randomly divided into two groups (gemcitabine therapy group and control group, *n*=6), and subjected to intraperitoneal injection of either gemcitabine (15 mg/kg) or saline (100 *μ*l, negative control) weekly. Xenograft tumors were measured every week using external calipers and their volumes were calculated based on the equation: V=(length × width^2^)/2. Five weeks later, mice were killed and tumor weight was measured, followed by IHC and TUNEL staining.

### Statistics

Data were presented as mean ±S.E.M. Group comparisons of normally distributed data were performed with unpaired Student’s *t*-test. For multiple comparisons, the Tukey–Kramer honestly significant difference was applied following ANOVA. Kaplan–Meier method and log-rank tests were used to determined cumulative survival and disease-free survival. The Pearson *χ*^2^ test was used to analyze the association of miR-3656 expression with *RHOF* expression. Correlations between miR-3656, RHOF and EMT marker expressions in 157 PC patients was evaluated by *χ*^2^ test. SPSS 17.0 software (IBM, Chicago, IL, USA) was used for all statistical analysis. Values of *P*<0.05 were considered statistically significant.
