# Supplementary material for: miR-3656 expression enhances the chemosensitivity of pancreatic cancer to gemcitabine through modulation of the RHOF/EMT axis
Source: Cell Death Dis. 2017 Oct 19;8(10):e3129–. doi: 10.1038/cddis.2017.530 (PMC5682692; doi:10.1038/cddis.2017.530)
Supplement: Supplementary Material [file cddis2017530x1.doc]

**Supplemental materials**

**Supplemental Table 1. Significant microRNA expression changes observed in gemcitabine-resistant PANC-1 cells**

|  | PANC-1 gemcitabine resistant cells | | | | | | |
| --- | --- | --- | --- | --- | --- | --- | --- |
| miRNA ID | Clone 1 | Clone 2 | Clone 3 | miRNA ID | Clone 1 | Clone 2 | Clone 3 |
|  | Down-regulation (Fold change) | | |  | Up-regulation (Fold change) | | |
| miR-23c | 0.07 | 0.10 | 0.01 | miR-4485 | 7.07 | 6.16 | 16.55 |
| miR-370-5p | 0.08 | 0.13 | 0.02 | miR-1261 | 4.18 | 11.30 | 9.84 |
| miR-3656 | 0.07 | 0.10 | 0.06 | miR-27a-5p | 4.52 | 10.99 | 6.63 |
| miR-487a-3p | 0.08 | 0.14 | 0.02 | miR-921 | 2.65 | 8.73 | 10.50 |
| miR-3146 | 0.14 | 0.06 | 0.22 | miR-363-3p | 9.83 | 7.50 | 3.60 |
| miR-494-5p | 0.18 | 0.17 | 0.11 | miR-1246 | 5.92 | 8.08 | 6.13 |
| miR-4659b-3p | 0.08 | 0.25 | 0.15 | miR-18b-5p | 4.52 | 11.81 | 3.39 |
| miR-3189-3p | 0.33 | 0.13 | 0.05 | miR-5095 | 3.78 | 4.01 | 11.58 |
| miR-20a-5p | 0.16 | 0.38 | 0.15 | miR-4743-5p | 3.72 | 3.24 | 9.12 |
| miR-19a-3p | 0.32 | 0.21 | 0.17 | miR-424-3p | 3.04 | 5.41 | 6.65 |
| miR-548at-5p | 0.49 | 0.04 | 0.19 | miR-20b-5p | 2.51 | 7.79 | 4.72 |
| miR-129-5p | 0.46 | 0.24 | 0.26 | miR-1255b-5p | 2.41 | 4.39 | 6.72 |
| miR-941 | 0.45 | 0.46 | 0.07 | miR-4704-5p | 4.34 | 6.59 | 2.57 |
| miR-4292 | 0.42 | 0.50 | 0.23 | miR-330-3p | 4.09 | 3.77 | 5.50 |
| miR-1538 | 0.39 | 0.45 | 0.32 | miR-122-3p | 3.42 | 6.28 | 2.86 |

**Supplemental Table 2. qPCR primers used in this study**

| Genes (*Homo sapiens*) | Primers | Sequences |
| --- | --- | --- |
| *RHOF* | Forward | 5'-AGAAGTACACGGCCAGCG-3' |
|  | Reverse | 5'-TTGTCGTAGCTGGTGGGATT-3’ |
| *E-cadherin* | Forward | 5'-CCGAGAGCTACACGTTCAC-3' |
|  | Reverse | 5'-CCGTAGAGGCCTTTTGACTG-3' |
| *N-cadherin* | Forward | 5'-GTATCCGGTCCGATCTGCA-3' |
|  | Reverse | 5'-ATAGTCCTGCTCACCACCAC-3' |
| *Vimentin* | Forward | 5'-CGGGAGAAATTGCAGGAGGA-3' |
|  | Reverse | 5'-TCCTCTTCGTGGAGTTTCTTCA-3' |
| *TWIST1* | Forward | 5'-GGGCCGGAGACCTAGATG-3' |
|  | Reverse | 5'-CCACGCCCTGTTTCTTTGAA-3’ |
| *CECR1* | Forward | 5'-GTTGGTGAGCTTTTCCGGTG-3' |
|  | Reverse | 5'-GAAACAGATGTGGCAGTGAGG-3' |
| *ATG9A* | Forward | 5'-GAGAGGAGCACATACTGTCCA-3' |
|  | Reverse | 5'-GTGACTTGCTCCCCTCGG-3' |
| *MRPL12* | Forward | 5'-TGAAGATCCAGGATGTCGGG-3' |
|  | Reverse | 5'-CAGCTTCACTTTGTCCACGG-3' |
| *LSP1* | Forward | 5'-TGGGGCCCAGACTACAGG-3' |
|  | Reverse | 5'-CCTGAAGCTGCCTGTCTCTC-3’ |
| *CHST12* | Forward | 5'-GTGAGGGTCGCGAGGTTC-3' |
|  | Reverse | 5'-AGGATCATGAACACCGACCC-3’ |
| *MNT* | Forward | 5'-ATAGAGACGCTACTGGAGGC-3' |
|  | Reverse | 5'-ACAGGAAGGGTATGTGCCAG-3' |
| *RPP30* | Forward | 5'-CGCTCACCTTGGCTATTCAG-3' |
|  | Reverse | 5'-GCAGTGAGATGGATCCGAGA-3' |
| *ALDH1B1* | Forward | 5'-GCCGGAACCAGAACCCAA-3' |
|  | Reverse | 5'-CAGAATGGGGCTTGGGAGG-3' |
| *NKRF* | Forward | 5'-ACCAACCACGTCTTCCTGG-3' |
|  | Reverse | 5'-CATGTTGAGAGGTGGCGTTT-3’ |
| *FNDC3B* | Forward | 5'-GTTGGAAGTAAAGAGGGTGCA-3' |
|  | Reverse | 5'-AGGCCACCTCGTAACTGTAG-3' |
| *EVI5* | Forward | 5'-TGCACAGAACAAAGGACTCC-3' |
|  | Reverse | 5'-TCTGGATTTCAAGCTCAGCA-3' |
| *KAT7* | Forward | 5'-GCGAAGGAAGAGGAATGCAG-3' |
|  | Reverse | 5'-AGTAAGCAGGCTCCTCAGTG-3' |
| *AIG1* | Forward | 5'-AGTGTTCTGGATCATTTATGCCT-3' |
|  | Reverse | 5'-TGGGATACTGATGGTGCGAT-3’ |
| *C1GALT1C1* | Forward | 5'-AACGTGAGAGGAAACCCGT-3’ |
|  | Reverse | 5'-CCATGACCAATCCTAATGTGTCC-3’ |
| *GAPDH* | Forward | 5'-GAAGGTGAAGGTCGGAGTC-3' |
|  | Reverse | 5'-GAAGATGGTGATGGGATTTC-3' |


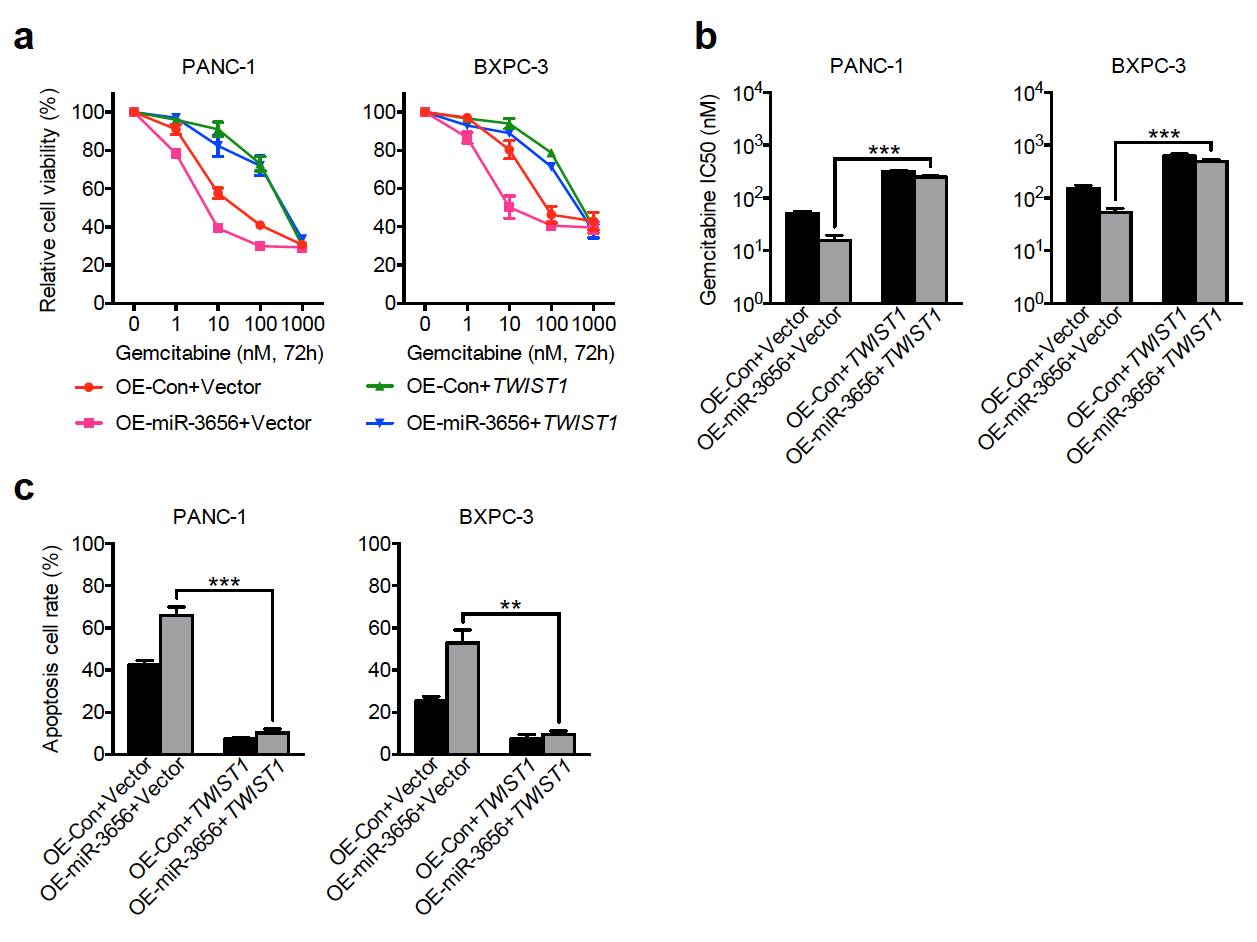


**Supplemental Figure 1. miR-3656 sensitization of PC cells to gemcitabine is TWIST1-dependent. (a-b)** Comparison of the relative cell viabilities and IC50s of gemcitabine between PC cells supplemented with miR-3656 mimic alone and cells transfected with both miR-3656 mimic and *TWIST1* vectors via MTS assay. **(c)** Apoptotic cell measurements in PC cells treated with miR-3656 mimic alone and cells transfected with both miR-3656 mimic and *TWIST1* vectors via flow cytometric analysis.
